# Phytochemical study of the plant species *Bidens pilosa* L. (Asteraceae) and *Croton floccosus* (Euphorbiaceae)

**DOI:** 10.12688/f1000research.112653.1

**Published:** 2022-06-27

**Authors:** Enrique Ruiz-Reyes, Mayte Ariana Mendoza-Cevallos, Ana Patricia Polanco-Moreira, Diego Germán Segovia-Cedeño, Ulbio Eduardo Alcivar-Cedeño, Alex Dueñas-Rivadeneira

**Affiliations:** 1 Departamento de Química Instituto de Ciencias Básicas, Universidad Técnica de Manabí, Avenida Urbina, y Che Guevara, Portoviejo, Manabí, 130105, Ecuador; 2 Universidad Técnica de Manabí, Avenida Urbina, y Che Guevara, Portoviejo, Manabí, 130105, Ecuador; 3 Departamento de Producción Animal, Facultad de Ciencias Zootécnicas, Universidad Técnica de Manabí, Portoviejo, Manabí, 130105, Ecuador; 4 Departamento de Procesos Químicos, Facultad de Ciencias Matemáticas, Físicas y Químicas, Universidad Técnica de Manabí, Portoviejo, Manabí, 130105, Ecuador; 5 Departamento de Procesos Agroindustriales, Facultad de Ciencias Zootécnicas, Universidad Técnica de Manabí, Portoviejo, Manabí, 130105, Ecuador

**Keywords:** extraction, phytochemical study, phenols, flavonoids, Bidens pilosa L, Croton floccosus, Asteraceae, Euphorbiaceae

## Abstract

**Background:** Given the chemical richness of medicinal plants ( *Bidens pilosa* L. and *Croton floccosus*) in Ecuador, they are considered the natural source of numerous medicines.

**Methods:** The leaves were dried at 40°C and 50°C and the extracts were characterized by means of phytochemical screening, verifying the presence of secondary metabolites such as alkaloids, reducing sugars, phenols, flavonoids, tannins and saponins. Three extraction processes were carried out, with two solvents of different polarities: hexane and ethanol. The extraction methods that were applied to the leaves of the plants were Soxhlet, ultrasonic bath and maceration, the latter two at room temperature and Soxhlet at the boiling temperature of the solvent. Determination of the total content of phenols and flavonoids is carried out using the Follin-Ciocalteau colorimetric reaction, Quercetin standard, Aluminum Chloride solution measured with a UV-Vis spectrophotometer. The antioxidant activity was performed with the DPPH radical and measured with the same equipment.

**Results**: The highest content of total phenols obtained by employing the Soxhlet method for extraction when the material was dried at 50°C was 48.609 ± 0.370 mg GAE/g of dry sample for *Bidens pilosa* L. while in the case of *Croton floccosus* it was 128.212 ± 0.601 mg GAE/g of dry sample obtained from the extraction by means of maceration. Finally, the antioxidant activity against the 1.1-diphenyl-2-picryl-hydrazyl radical was determined, and it was found that the *Bidens pilosa* L. species performed better and responded better to the test, with an IC _50_ value of 239.33 µg/mL, than *Croton floccosus* (IC _50_ of 644.125 µg/mL).

**Conclusions: **The following preliminary phytochemical study of the *Bidens pilosa* L. and *Croton floccosus* plants provided important information on the content of secondary metabolites and response to the DPPH radical reported for the first time in Ecuador, which may be future use for medicinal application.

## Introduction

The biodiversity of Ecuador ranks sixth worldwide for the number of species. ^1^ Many species comprise the *Croton* genus in this country, and 39 have been recognized. ^2^ Of these, 13 are considered native, ^3^ and the others have been documented in the last 15 years. ^4^^,^^5^ Many species of this genus have been used in traditional medicine by the country’s indigenous population. Antimicrobial, ^6^ antioxidant, ^7^ anti-inflammatory ^8^ and anticancer ^9^^,^^10^ biological activity studies have been carried out on them in America, Asia and Africa.

Brian A. Smith found a new species of *Croton* (Euphorbiaceae) on the western slopes of the Ecuadorian Andes in 2006, *Croton floccosus.* ^11^ This is described as a medium-sized tree with grayish external and internal bark with a reddish exudate, along with simple leaves (4–15 × 2.5–8 cm) and alternate elliptic-oval unisexual flowers and tricoco-type fruits with persistent styles. This plant is commonly found next to streams and disturbed sites in the provinces of Pichincha and Imbabura in Ecuador.

Some 230 to 240 species of *Bidens* have been described, ^12^^,^^13^ including *Bidens pilosa*, which was identified in 1753 by Carl Linnaeus. This is a representative perennial herb, distributed globally in temperate and tropical regions, which has traditionally been used in food and medicine without having any obvious adverse effects. About 116 publications on this species have documented its medical use, ^14^ and phytochemical studies show that it contains a high number of flavonoids and polyines, which have anticancer, ^15^ anti-inflammatory, ^16^ antibacterial ^17^ biological activity, as well as antifungal, ^18^ antimalarial, ^19^ and antioxidant ^20^^,^^21^ properties.

*Bidens pilosa* is an erect, strongly branched plant with a strong aromatic odor. It has opposite leaves and a long petiole, whose limbs are generally deeply divided into 3 to 5 segments giving it the appearance of a compound leaf. It also has white ray flowers and numerous yellow tubular flowers. ^13^

Although this species has been studied to a greater extent than *Croton floccosus*, we have not found phytochemical studies of these plants in Ecuador, which is why it has important research potential that may allow us to transform traditional knowledge into scientific knowledge.

In the following work, a preliminary phytochemical study of the leaves of the species *Bidens pilosa* and *Croton floccosus* was carried out using the Tukey test, ^22^ which is employed in ANOVA in order to compare the means of the values obtained. This study joins the recently published work by Ruiz-Reyes *et al*. on *Melampodium divaricatum* and *Zanthoxylum sprucei* plants, ^23^ in an attempt to discover which active compounds are present and if they have the potential to be used as medicinal plants.

## Methods

### Vegetal material

The fresh leaves of the *Bidens pilosa* L. and *Croton floccosus* species were collected in January 2021 from the Botanical Garden at the Technical University of Manabí (UTM), Portoviejo, in the Manabí province in western Ecuador. The botanical identification was carried out by the botanists in charge of the herbarium in the botanical garden at the UTM. The vouchers of the specimens were deposited with the following codes *Bidens pilosa* L. (Asteraceae) and *Croton floccosus* (Euphorbiaceae).

### Chemicals and reagents

The chemicals and reagents employed in this study were: Folin-Ciocalteu reagent, 1.1-diphenyl-2-picryl-hydrazyl (DPPH), sodium nitrite, aluminum chloride, sodium hydroxide, sodium carbonate, catechin, quercetin, trolox, methanol, and ethanol. All the reagents and solvents were supplied by Sigma-Aldrich and are of an appropriate analytical grade for study.

### Extraction

The plant material was dried in a tray dryer (universal oven UF110, Memmert) at temperatures of 40°C and 50°C. A reference weight was taken every two hours until it remained constant. The material was then allowed to finish drying, after which it was pulverized. The extracts of the crude plants were obtained using the ultrasonic bath, maceration and Soxhlet extraction methods. In the case of Soxhlet extraction, 10 g of plant material were used and placed in the equipment extractor cartridge with 250 mL of ethanol. Between 8 and 10 extractions were performed at the boiling temperature of the solvent, and the solvent was eventually evaporated in order to obtain the extracts. With regard to ultrasonic bath extraction, 10 g of finely divided plant material was placed in a 250 mL beaker containing 100 mL of ethanol. The mixture was sonicated at 50 W for 1 h in an ultrasonic bath and subsequently filtered. The solvent was then evaporated in a vacuum to obtain the corresponding extracts for analysis. In the case of extraction with maceration, 10 g of finely divided plant material was placed in a 250 mL beaker with 100 mL of ethanol. The mixture was macerated for 72 hours and stirred from time to time. It was subsequently filtered and the solvent was evaporated in a vacuum to obtain the corresponding extracts for analysis.

### Phytochemical screening

The crude extracts were evaluated phytochemically to determine the presence of chemical constituents using standard procedures, following the same methodology described by Ruiz-Reyes ^23^^,^^24^ for the determination of metabolites: Alkaloids: Each extract (10 mg) was dissolved in 2 mL of 5% hydrochloric acid and, after mixing and filtering, three aliquots were taken. Drops of Wagner, Mayer, Bouchardat, and Dragendorff reagents were added to each one. A red-brown precipitate (Wagner), a yellowish white precipitate (Mayer), a brown precipitate (Bouchardat), and an orange-red precipitate (Dragendorff) indicated the presence of these metabolites. Flavonoids (Shinoda test): 1 ml of absolute ethanol and three drops of concentrated hydrochloric acid were added to 10 drops of the extract diluted in isopropyl alcohol. The formation of a red color indicated the presence of aurones and chalcones while the formation of an orange, red, or magenta color formation indicated the presence of isoflavones, flavonols, and flavonoids, respectively. Saponins (with sodium bicarbonate): 1 mL of distilled water and one drop of saturated sodium bicarbonate solution were added to five drops of the extract dissolved in isopropyl alcohol (20 mg/mL) in a test tube and shaken vigorously for three minutes. The formation of honeycomb shaped foam indicated the presence of saponins, and tannins: 10 mg of each extract was dissolved in 1 mL of ethanol and extract with 3 mL of boiling distilled water for 15 minutes. Once it had been allowed to stand at room temperature, 0.2 mL of 10% sodium chloride solution was added to the mixture and filtered. In addition, four drops of 10% ferric chloride solution were added. The precipitation observed was indicative of the presence of tannins. For reducing sugars, 200 μL of the extract were dissolved in a tube, Benedict’s reagent was added until the extract turned bluish, it was then heated in a water bath at a temperature of 60°C. It was considered positive if it turned reddish brown. ^25^

### Determination of total phenols and flavonoids

Determination of the total content of phenols in the extracts was carried out using the Follin-Ciocalteau reaction and by employing gallic acid as the reference phenolic compound. The gallic acid calibration curve was prepared by weighing 2 mg of gallic acid and making it up to a volume of 10 mL with distilled water, which is the stock solution at a concentration of 0.2 mg/mL. Aliquots of 50 μL were subsequently taken, after which 200 μL of Follin-Ciocalteau reagent and 2 mL of 7% Na _2_CO _3_ were added, and the solution was made up to 5 mL. After the absence of light for 30 minutes and at room temperature, we measured the absorbance at 725 nm using the prepared solution as a blank without the analyte. Total flavonoid content was determined using quercetin as standard. For the preparation of the Quercetin standard, 2 mg of the Quercetin standard was weighed and dissolved with 70% methanol in a 10 mL volumetric flask, which is considered the mother solution with a concentration of 0.2 mg/mL. The aluminum chloride solution was subsequently prepared by weighing 500 mg of AlCl _3_ and dissolving it with a solution of 25 mL 5% Acetic Acid in methanol, which led to a solution with a concentration of 20 mg/mL and aluminum chloride. In order to perform the calibration curve with the Quercetin standard, aliquots of 5, 10, 15, 20, 25, 30, 35, 40 μL of the standard solution were taken, and made up to 1 mL with methanol (70%), after which 1 mL of the AlCl _3_ solution was added. The same solution was used as a blank without adding the standard compound. After waiting 15 min for the reaction, the absorbance of the solution was measured at a wavelength of 430 nm in the UV-Vis spectrophotometer. The preparation of the extracts was carried out by taking 5 mg of the sample, which was dissolved in 5 mL of 70% aqueous methanol. The determination procedure for total flavonoids was carried out by taking 200 μL aliquots of different extracts of the sample in triplicate and making them up with 1 mL of methanol (70%), after which 1 mL of the AlCl3 solution was added. After waiting 15 min, the absorbance of the solution was measured at a wavelength of 430 nm in the UV-Vis spectrophotometer. Both methodologies were described by Ruiz-Reyes. ^23^^,^^26^^–^^28^

### Test with the radical DPPH

The antioxidant activity was determined using a spectrophotometer and the DPPH molecule as a reagent to generate the free radical following the methodology described by the author himself and other authors. ^23^^,^^29^ DPPH reagent preparation: 0.02 g of the DPPH reagent was weighed out and made up to volume with 100 mL of methanol in a flask. This was then homogenized and left to react for 24 hours in an amber bottle in the dark.

• Wavelength determination
  2.5 mL of the prepared DPPH reagent was taken and 15 mL ethanol added. A scan was carried out in the spectrophotometer. A maximum absorbance of 0.750 ± 0.05 should appear at a wavelength of 517 nm.
• Blank preparation
  Methanol (15 mL) was added and a scan was performed in the spectrophotometer to verify that it did not have absorbance in the working wavelength range in which the maximum of the sample is found.
• Sample preparation
  We took 3.0 mL of the DPPH reagent and 100, 200, 300, 400, 500 μL of the extract added. Once the sample and the blank were prepared, the absorbance reading was performed in the Thermo Scientific Genesys 180 (UV-Visible) spectrophotometer at 517 nM every 10 minutes.

## Results and discussion

### Analysis of extraction methods (yield)

A multifactorial statistical analysis of variance (ANOVA) was carried out for the different species studied, with a maximum order of interaction of 2 for the yield values. This made it possible to determine which of the extraction factors evaluated had a statistically significant effect on the yield. Table 1 presents a summary of the multifactorial arrangement used for the processes of obtaining extracts from *Bidens pilosa* L. and *Croton floccosus* at drying temperatures of 40 and 50°C.

**Table 1.** Yields of *Bidens pilosa* and *Croton floccosus* at drying temperatures of 40 and 50°C.

*Bidens pilosa* L.				*Croton floccosus*			
A: Drying temperature (°C)	B: Extraction process	C: Solvent	Yield (%)	A: Drying temperature (°C)	B: Extraction process	C: Solvent	Yield(%)
40	Soxhlet	Ethanol	22.04	40	Soxhlet	Ethanol	38.08
	Maceration		24.96		Maceration		12.5
	Ultrasonic bath		37.95		Ultrasonic bath		33.8
	Soxhlet	Hexane	13.26		Soxhlet	Hexane	3.57
	Maceration		0.96		Maceration		4.2
	Ultrasonic bath		4.77		Ultrasonic bath		4.48
50	Soxhlet	Ethanol	55.55	50	Soxhlet	Ethanol	50.63
	Maceration		23.46		Maceration		14.26
	Ultrasonic bath		54.52		Ultrasonic bath		33.48
	Soxhlet	Hexane	6.24		Soxhlet	Hexane	0.79
	Maceration		3.5		Maceration		3.2
	Ultrasonic bath		3.5		Ultrasonic bath		0.7

Table 2 shows the analysis of variance for the extraction performance in the case of *Bidens pilosa* L., for which it was determined that there is a statistically significant difference in the treatments. The contribution of each factor was measured by eliminating the effects of the other factors. The P-values test the statistical significance of each of the factors. Since the P-value of factor C (solvent) is less than 0.05, this factor has a statistically significant effect on performance at a confidence level of 95.0%.

**Table 2.** Analysis of variance for extraction yields of *Bidens pilosa* L.

Source	Sum of squares	Gl	Middle square	F-Reason	P-Value
MAIN EFFECTS					
A: Drying temperature	152.867	1	152.867	1.23	0.3827
B: Extraction process	354.869	2	177.434	1.43	0.4117
C: Solvent	2890.76	1	2890.76	23.28	0.0404
INTERACTIONS					
AB	81.3555	2	40.6778	0.33	0.7532
AC	245.979	1	245.979	1.98	0.2946
BC	208.387	2	104.194	0.84	0.5437
WASTE	248.338	2	124.169		
TOTAL (CORRECTED)	4182.55	11			

**Figure 1.**
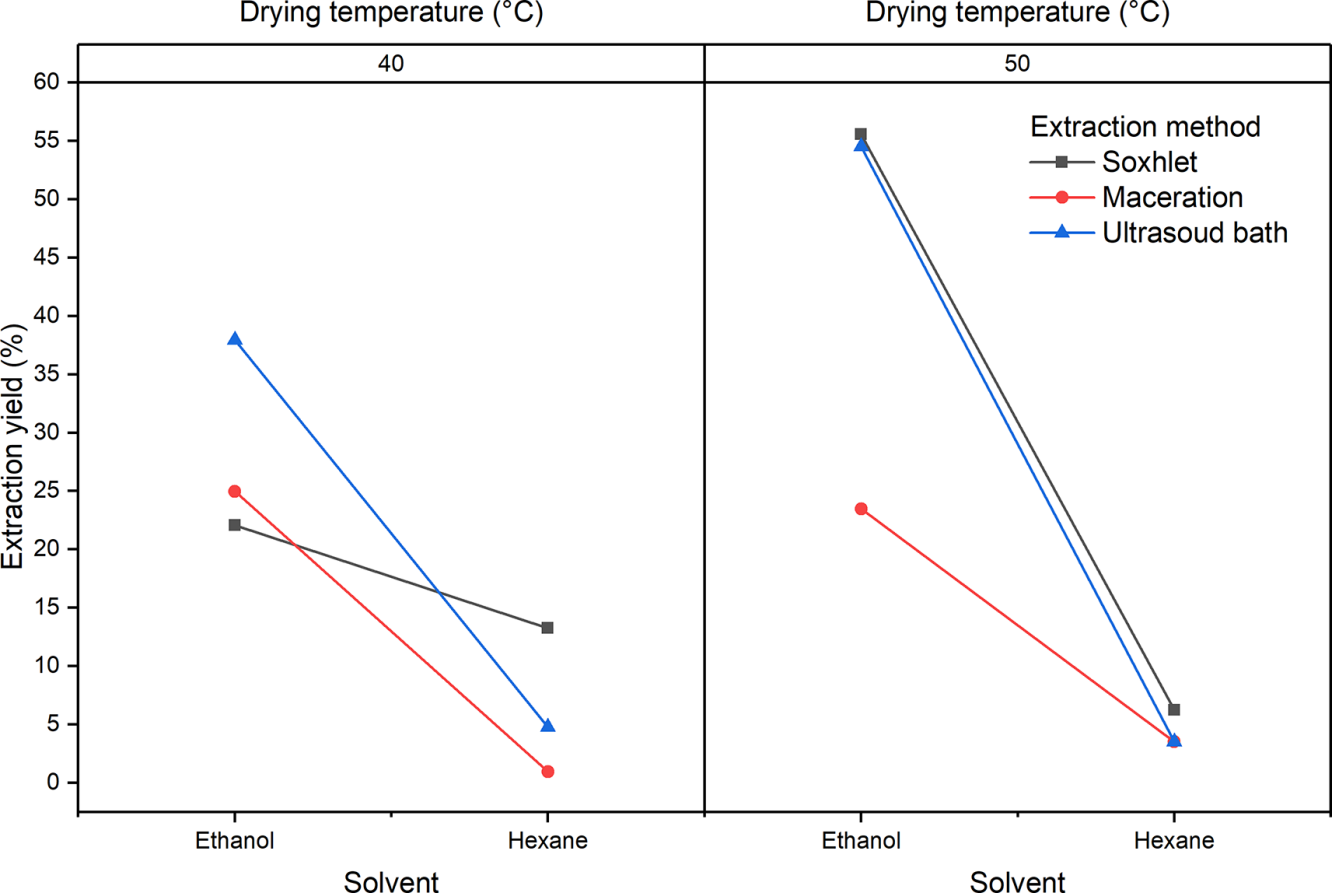
ANOVA graph for the influence of factors on the extraction performance of *Bidens pilosa* L.

Figure 1 shows the influence of the factors of the experimental design with respect to the extraction yield for the extracts obtained from *Bidens pilosa* L. A notable decrease in yields is observed when the extraction process is carried out using hexane. Although it is not statistically significant, it is necessary to mention that there was a better yield in the extraction processes in which leaves dried at 50°C were used. The process that obtained the best yields was the Soxhlet extraction.

For each significant factor, multiple range tests provide information regarding which means were significantly different from others. Tukey’s honestly significant difference (HSD) method was selected for this test because it allows the comparison of individual means from an analysis of variance of several samples subjected to different treatments, thus widening the intervals in order to allow multiple comparisons between all pairs of means.

**Figure 2.**
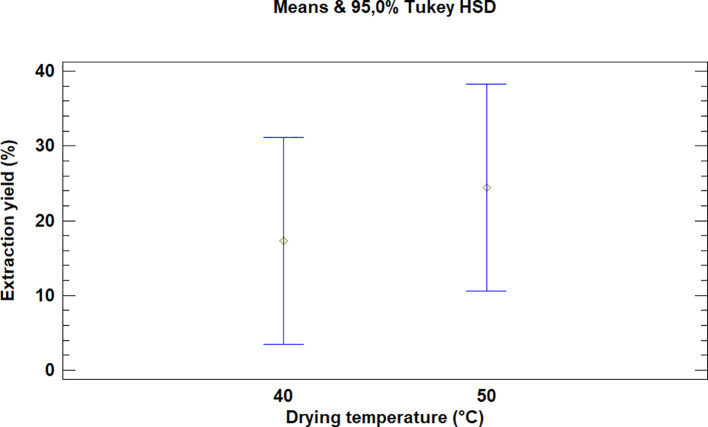
Comparison of Tukey's means for yields of *Bidens pilosa* L. extracts according to drying temperature.

This test showed that there was no significant difference between the means for the drying temperatures of the leaves of *Bidens pilosa* L., as is noted in Figure 2. It is necessary to mention that although there was no statistically significant difference between the temperatures used for drying, the extraction processes during which 50°C was used led to higher yield values.

**Figure 3.**
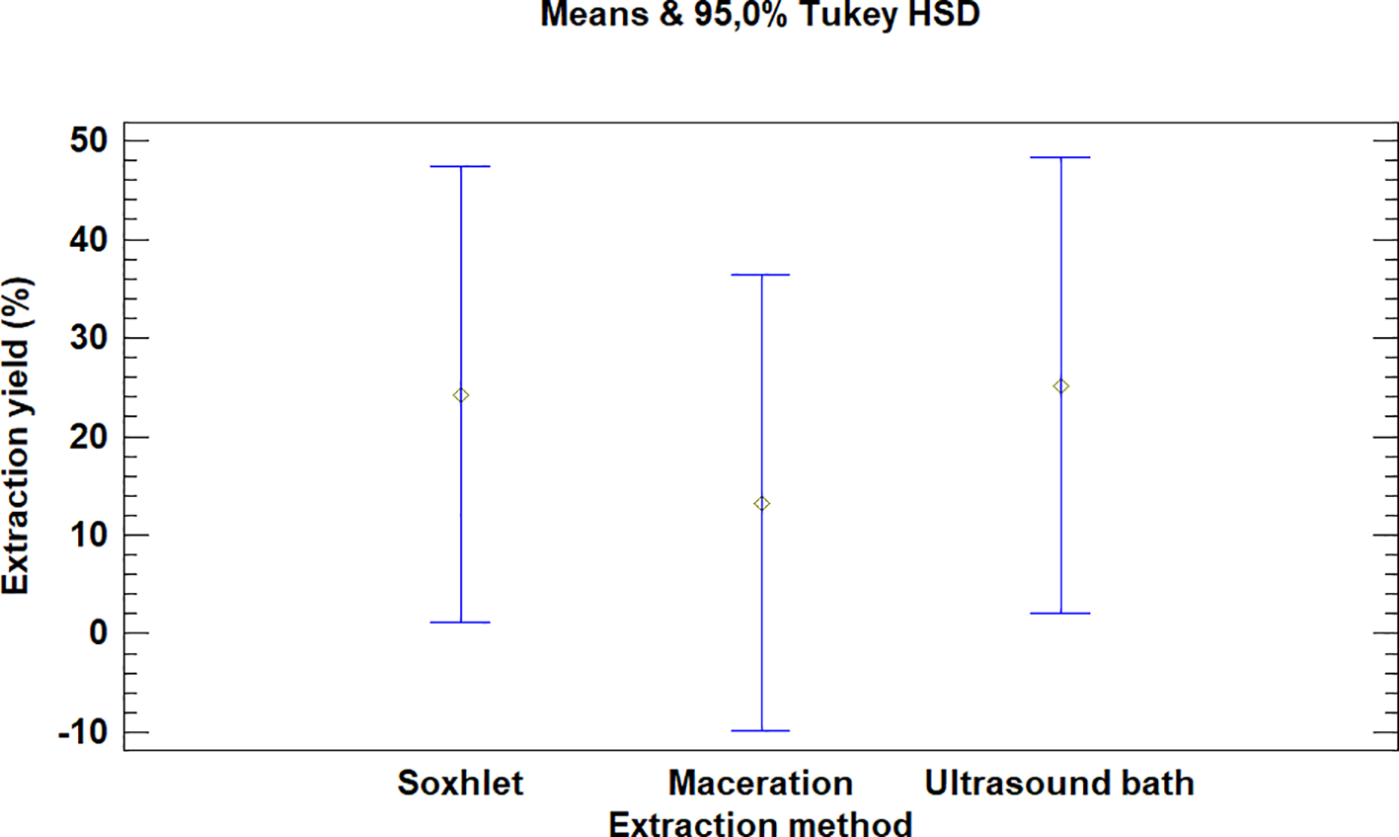
Comparison of Tukey means for yields of *Bidens pilosa* L. extracts according to the extraction process.

The multiple range test carried out for *Bidens pilosa* L. also showed that there was no difference between the extraction process methods evaluated, as is noted in Figure 3. It is, however, necessary to mention that the extraction with an ultrasonic bath and Soxhlet led to higher yields than the maceration process, despite the fact that they are not statistically different.

Furthermore, Table 3 shows the analysis of variance for the extraction performance of *Croton floccosus*, which determined that there is a statistically significant difference in the treatments. The contribution of each factor is measured by eliminating the effects of the other factors. The P-values test the statistical significance of each of the factors. Since the P-value of factor C (solvent) is less than 0.05, this factor has a statistically significant effect on the extraction yield for *Croton floccosus* at a confidence level of 95.0%. It is also necessary to highlight that the interaction between factor B (extraction process) and C (solvent) has a significant effect on the extraction process.

**Table 3.** Analysis of variance for extraction yields of *Croton floccosus.*

Source	Sum of squares	Gl	Middle square	F-Reason	P-Value
MAIN EFFECTS					
A: Drying temperature	3.44541	1	3.44541	0.28	0.6517
B: Extraction process	446.838	2	223.419	17.91	0.0529
C: Solvent	2291.08	1	2291.08	183.66	0.0054
INTERACTIONS					
AB	24.7647	2	12.3824	0.99	0.5019
AC	38.7002	1	38.7002	3.10	0.2202
BC	545.456	2	272.728	21.86	0.0437
WASTE	24.9493	2	12.4747		
TOTAL (CORRECTED)	3375.23	11			

**Figure 4.**
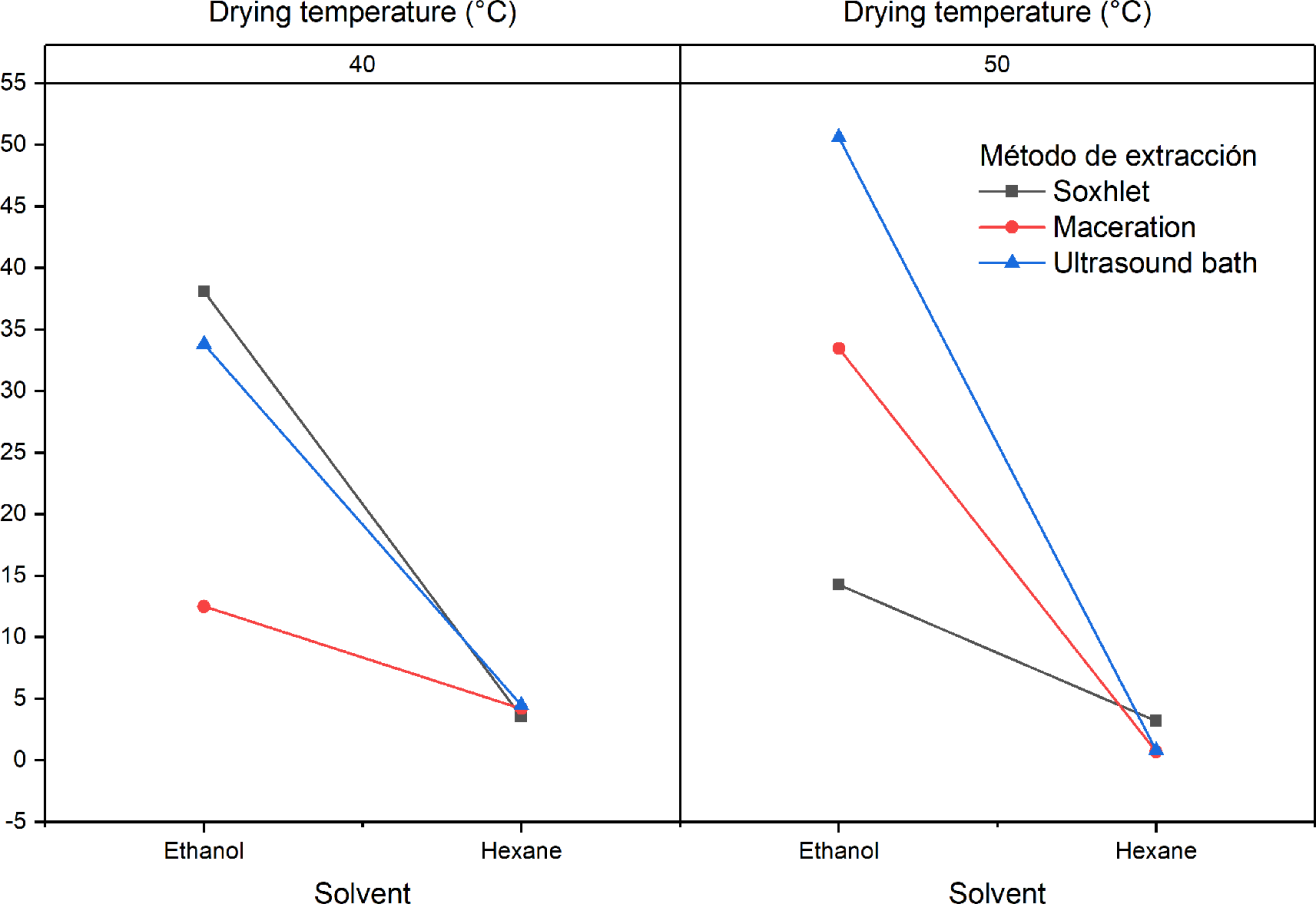
ANOVA graph for the influence of factors on the extraction yield of *Croton floccosus.*

Figure 4 shows the influence of the factors of the experimental design on the extraction yield. A notable decrease in yields is observed when hexane is used in the extraction process. It will also be observed that there is a difference with respect to the yields obtained as regards the interaction between the extraction method and the solvent (B and C), showing that the highest yields are obtained with the Soxhlet extraction processes with ethanol at both drying temperatures.

**Figure 5.**
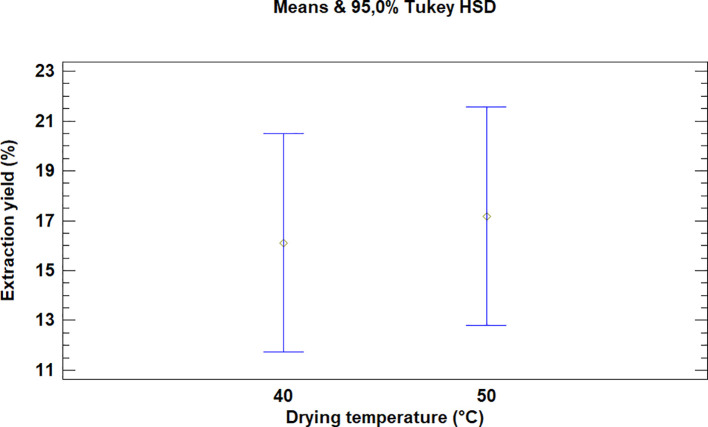
Comparison of Tukey means for yields of *Croton floccosus* extracts according to drying temperature.

As described above for the temperatures selected in order to dry *Bidens pilosa* L., there is no significant difference between the means obtained for the drying temperatures of *Croton floccosus* leaves ( Figure 5). However, it is necessary to mention that, although there is no statistically significant difference, the extraction processes during which the drying temperature of the material was 50°C obtained better extraction yields, as occurred with the *Bidens pilosa* L.

**Figure 6.**
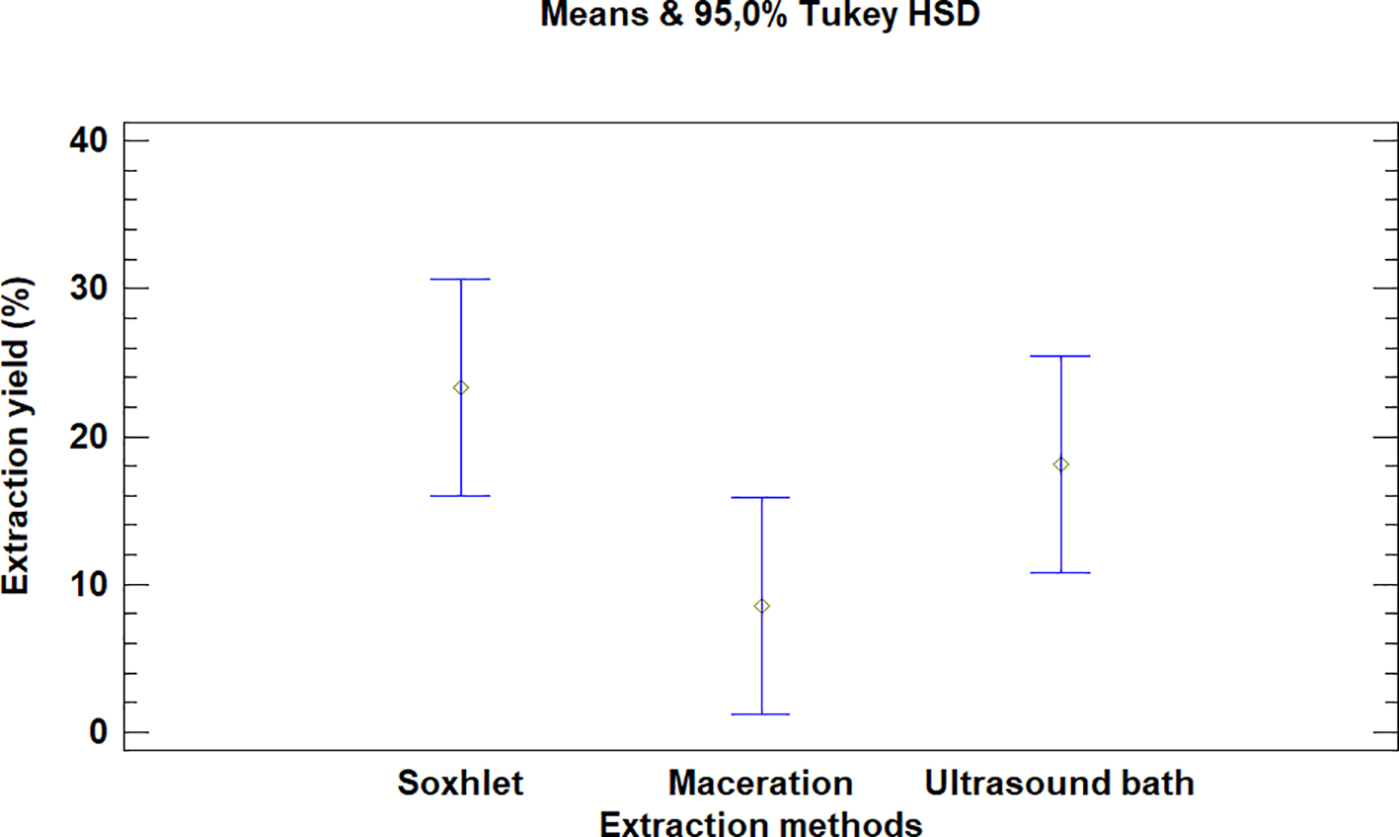
Comparison of Tukey means for yields of *Croton floccosus* extracts according to the extraction method.

It was also noted that the multiple range test carried out for *Croton floccosus* made it possible to identify that there was a difference between the Soxhlet and maceration process extraction methods ( Figure 6). The Soxhlet extraction process method obtains higher yields than the maceration and ultrasonic bath extraction methods.

### Analysis for solvents

**Figure 7.**
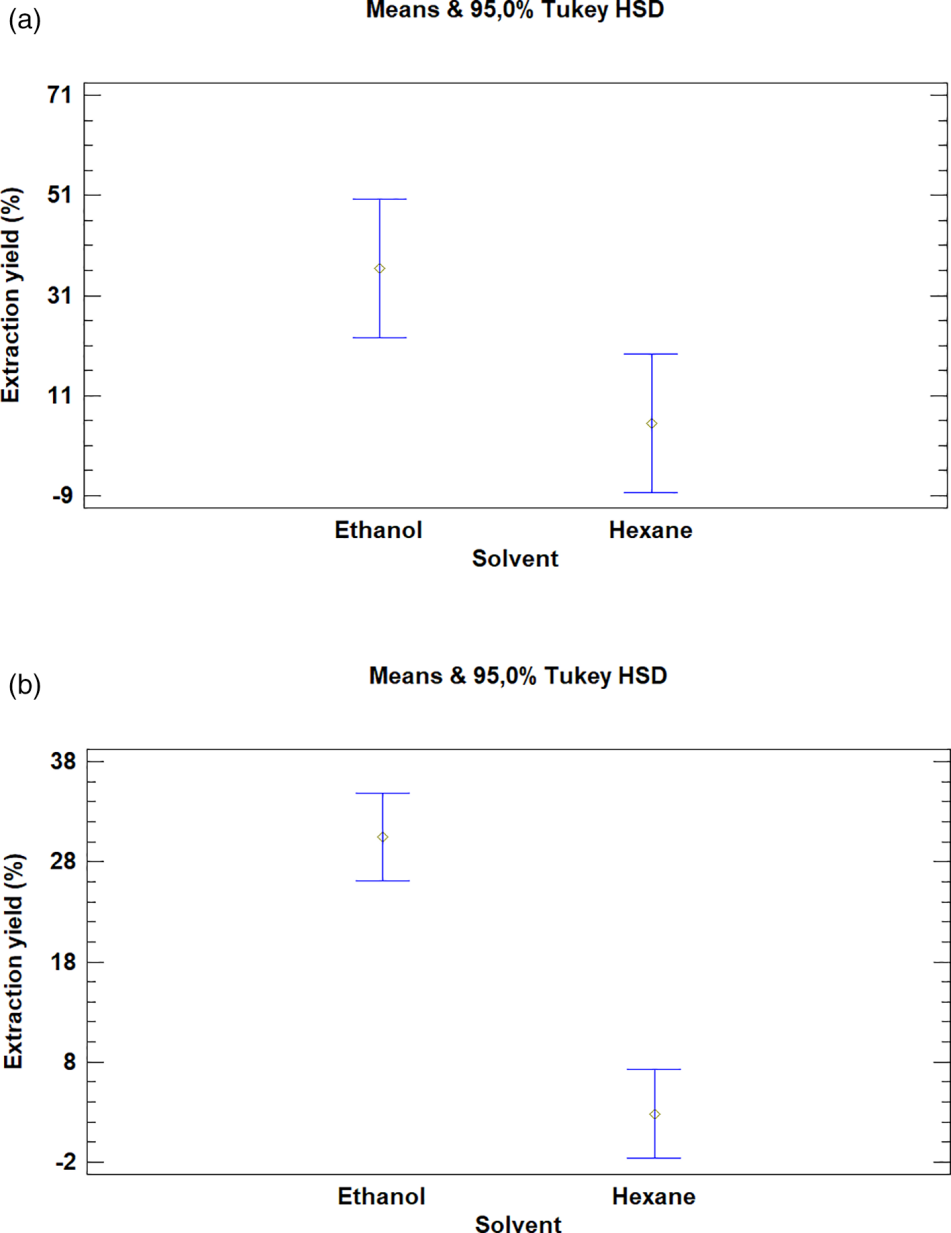
Comparison of Tukey means for yields of extracts of *Bidens pilosa* L. and *Croton floccosus* according to the solvent used.

Finally, in the case of both plants, the multiple range test showed that there was a difference between the solvents used in the extraction ( Figure 7). The processes in which hexane was used obtained a low yield when compared to extractions with ethanol.

### Data obtained from phytochemical tests

Table 4 details the qualitative results of the phytochemical tests performed on the ethanolic extracts of *Bidens pilosa* L. (Asteraceae) and *Croton floccosus* (Euphorbiaceae), obtained with Soxhlet extraction. There is a low presence of flavonoids and saponins, and a relatively abundant presence of phenols and tannins for both species. In addition, a relatively abundant presence of alkaloids is detected for the species *Croton floccosus* and no reducing sugars are detected, while a low presence of reducing sugars is detected for the species *Bidens pilosa* L.

**Table 4.** Results of the phytochemical tests carried out on the ethanolic extracts of the species *Bidens pilosa* L. and *Croton floccosus* obtained with Soxhlet extraction.

	Alkaloids	Reducing sugars	Phenols	Flavonoids	Tannins	Saponins
Ethanolic extract of *Bidens pilosa* L. obtained by Soxhlet	+	+	+ +	+	+ +	+
Ethanol extract of *Croton floccosus* obtained by Soxhlet	+ +	-	+ +	+	+ +	+

The equivalent mg of gallic acid corresponding to each gram of dry sample of *Bidens pilosa* L. were obtained, as shown in ( Table 5). As will be noted, Tukey’s HSD test for the phenolic content of the different samples made it possible to determine that there are significant differences among them (p<0.05). Samples that share the same letter are homogeneous.

**Table 5.** Total phenolic content corresponding to the equivalent of gallic acid.

Sample	mg gallic acid equivalent/g dry sample			Average ± D. standard
	1	2	3	
Soxhlet at 40°C	38.546	39.036	39.444	39.009 ± 0.450 ^a^
Soxhlet at 50°C	48.192	48.738	48.898	48.609 ± 0.370 ^b^
Ultrasonic bath at 40°C	30.73	30.35	29.734	30.271 ± 0.503 ^c^
Ultrasonic bath at 50°C	40.256	40.302	40.546	40.368 ± 0.156 ^d^
Maceration at 40°C	26.446	27.092	27.566	27.035 ± 0.562 ^e^
Maceration at 50°C	29.746	30.428	30.748	30.307 ± 0.512 ^c^

Values are the mean of triplicate determinations. Different lowercase letters indicate statistical difference p<0.05.

Based on data from Ruiz Reyes *et al*. ^30^

**Figure 8.**
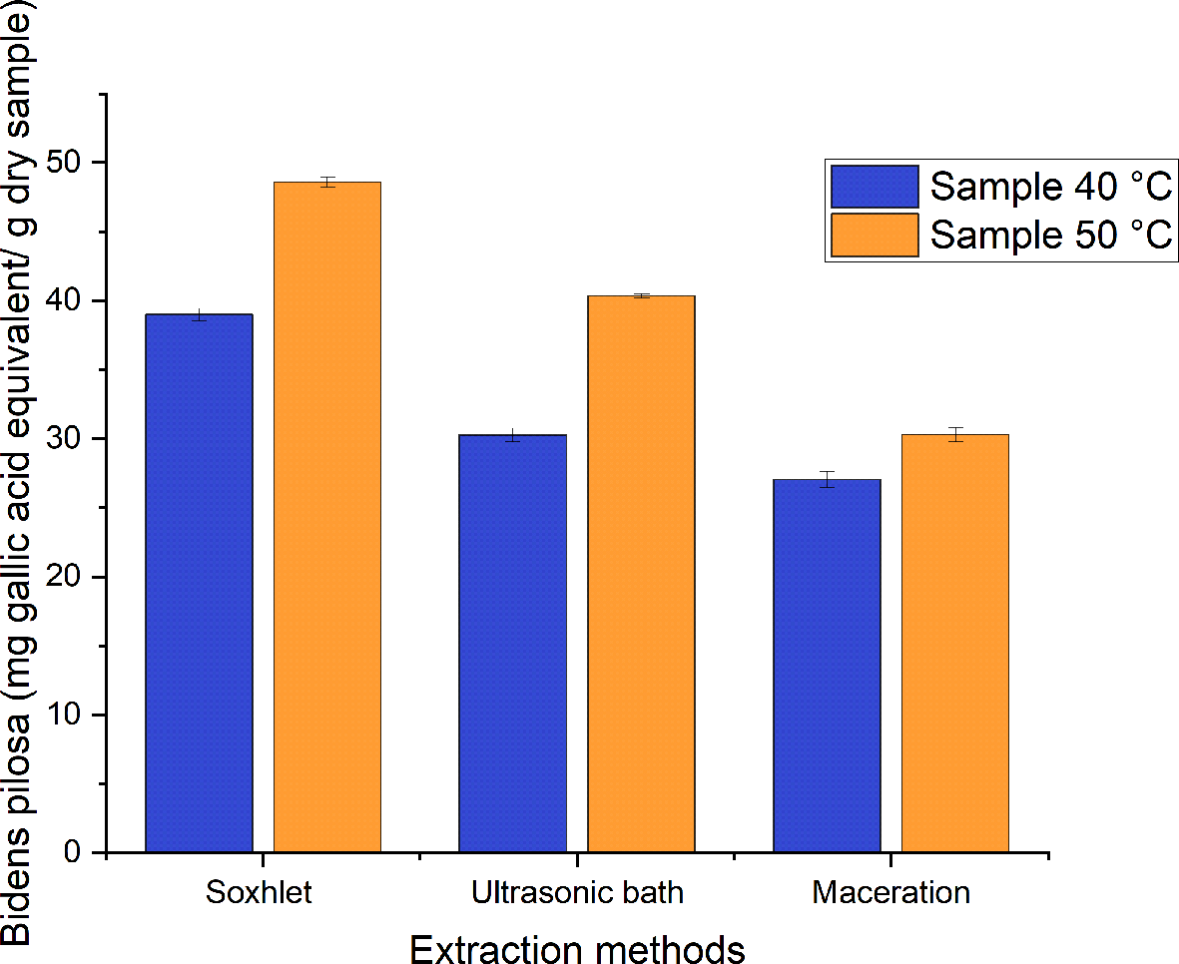
Phenolic content of the different ethanolic extracts of *Bidens pilosa* L.

As can be seen in Figure 8, the phenolic content for the extracts has values of 27–49 mg GAE/g of dry sample for the different processes employed to obtain ethanolic extracts of *Bidens pilosa* L. with leaves dried at 40°C and 50°C. Those with the lowest value correspond to the extracts obtained using the maceration method and those with the highest value correspond to the Soxhlet extraction process, with a value of 48.609 ± 0.370 mg GAE/g of dry sample of the extract obtained from the material dried at 50°C. In their research, Singh *et al*. ^31^ obtained methanolic extracts from leaves of *Bidens pilosa* L. dried at 30°C with a total phenolic content corresponding to 72 mg GAE/g of dry extract. This value is approximately 32% higher than that obtained in the present investigation, but it is necessary to mention that the extraction carried out by Singh *et al*. was carried out using an extraction process involving broken evaporation at a reduced pressure. In their investigation, meanwhile, Falowo *et al*. ^32^ obtained phenolic content for the extracts of *B. pilosa* corresponding to 75.9 mg GAE/g of dry extract when an exhaustive maceration was carried out with an ethanol-water solution (7:3) with shaking at room temperature for two days.

The equivalent mg of gallic acid corresponding to each gram of dry *Croton floccosus* sample were obtained in the same way. These values are presented in Table 6. It was noted that Tukey’s HSD test for the phenolic content of the different samples determined that there is a significant difference among them (p <0.05) Samples that shared the same letter are homogeneous.

**Table 6.** Total phenolic content corresponding to the equivalent of gallic acid.

Sample	mg gallic acid equivalent per g of dry sample			Average ± D. standard
	1	2	3	
Soxhlet at 40°C	124.146	125.562	125.55	125.086 ± 0.814 ^a^
Soxhlet at 50°C	89.86	89.746	89.9	89.835 ± 0.080 ^b^
Ultrasonic bath at 40°C	42.614	42.964	42.988	42.855± 0.209 ^c^
Ultrasonic bath at 50°C	80.576	80.618	80.82	80.671 ± 0.130 ^d^
Maceration at 40°C	121.91	122.284	121.704	121.966 ± 0.294 ^e^
Maceration at 50°C	127.874	127.856	128.906	128.212 ± 0.601 ^f^

Values are the mean of triplicate determinations. Different lowercase letters indicate statistical difference p<0.05.

Based on data from Ruiz Reyes *et al*. ^30^

**Figure 9.**
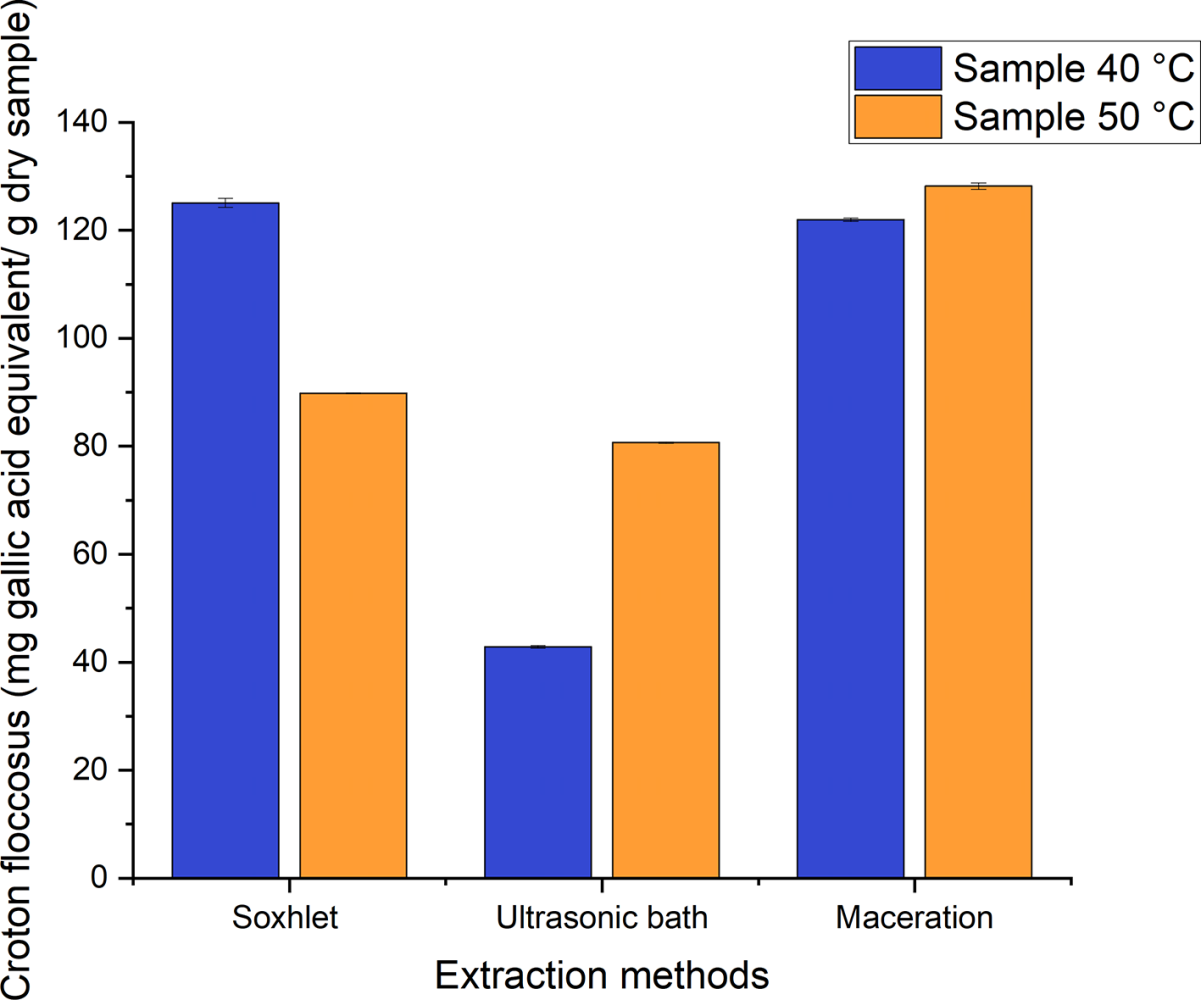
Phenolic content of the different ethanolic extracts of *Croton floccosus.*

A range of 42–129 mg GAE/g of dry sample were obtained from the different processes employed to obtain ethanolic extracts of *Croton floccosus*, as can be seen in Figure 9. Those with the lowest value correspond to the extracts obtained using the ultrasonic bath method, and those with the highest value correspond to the extraction process carried out using maceration, with a value of 128.212 ± 0.601 mg GAE/g of dry sample of the extract obtained from the material dried at 50°C. This contrasts strongly with the values obtained for *Bidens pilosa* L., since the extracts obtained by means of maceration had the lowest total phenolic content. Taking into account that this species is considered endemic to Ecuador, Pazmiño, ^33^ in his research, obtained ethanolic extracts from the freeze-dried “latex” of *Croton floccosus*, which had a total phenolic content corresponding to 271.212 ± 3.1728 mg GAE/g of dry extract. This value is approximately 53% higher than that obtained in the present investigation, and it is necessary to mention that the values presented by this author correspond to those of an extract obtained from the “latex” of this species.

### Content of total flavonoid tests

The equivalent mg of quercetin corresponding to each gram of dry sample of *Bidens pilosa* L. were obtained, as shown in Table 7. Tukey’s HSD test for the flavonoid content of the different samples determined that there is a significant difference among them (p<0.05). Samples that shared the same letter are homogeneous.

**Table 7.** Total flavonoid content corresponding to quercetin equivalents.

Sample	mg quercetin equivalent/g dry sample			Average ± D. standard
	1	2	3	
Soxhlet at 40°C	17.851	17.809	17.725	17.795 ± 0.0644 ^a^
Soxhlet at 50°C	16.879	16.815	16.853	16.849 ± 0.0322 ^b^
Ultrasonic bath at 40°C	17.643	17.705	17.648	17.665 ± 0.0042 ^a^
Ultrasonic bath at 50°C	16.583	16.590	16.582	16.585 ± 0.0218 ^b^
Maceration at 40°C	13.966	14.009	13.982	13.986 ± 0.0218 ^c^
Maceration at 50°C	12.374	12.849	12.440	12.554 ± 0.2573 ^d^

Values are the mean of triplicate determinations. Different lowercase letters indicate statistical difference p<0.05.

Based on data from Ruiz Reyes *et al*. ^30^

**Figure 10.**
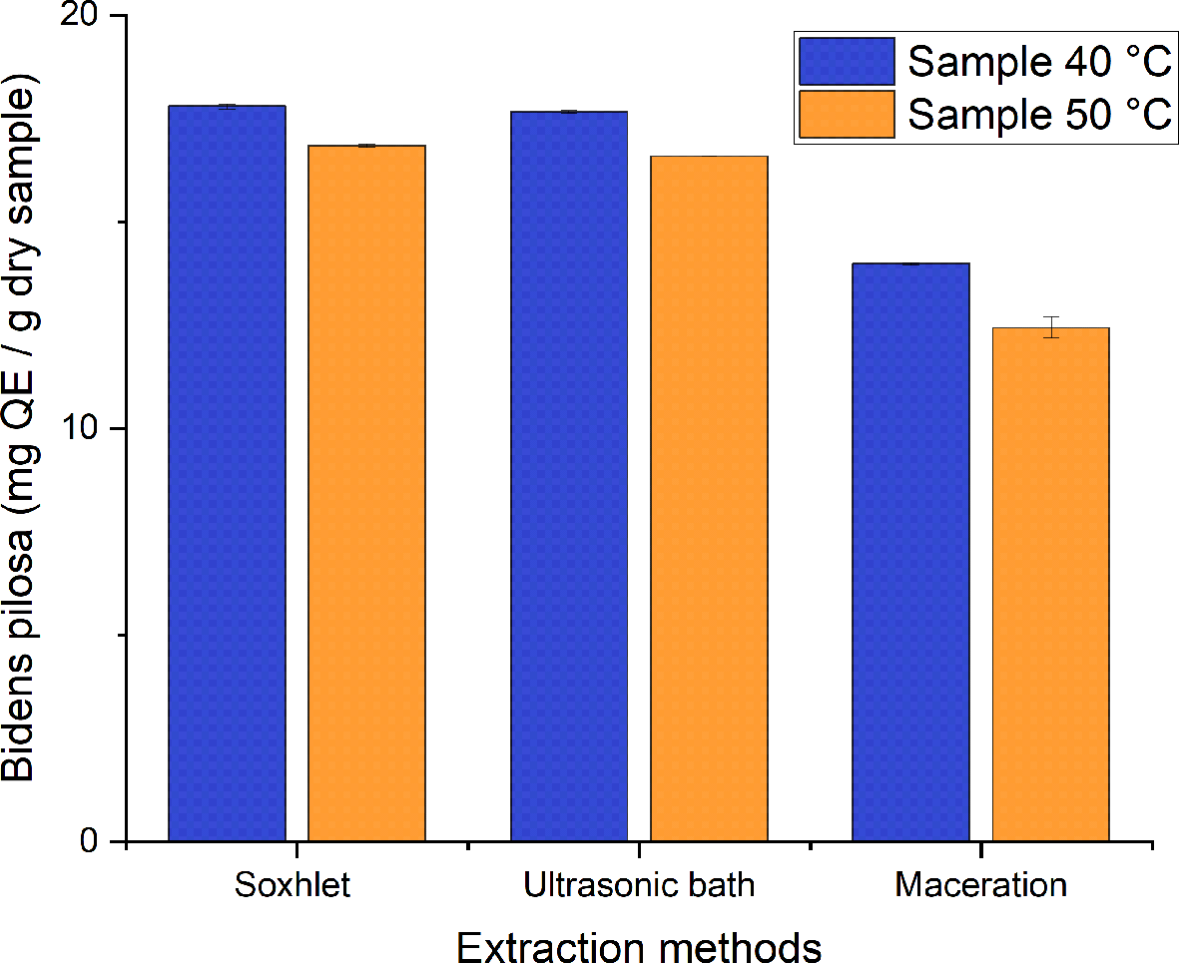
Total flavonoid content of the different ethanolic extracts of *Bidens pilosa.*

A range of 12–18 mg QE/g of dry sample were obtained from the different processes employed to obtain ethanolic extracts of *Bidens pilosa* L., as can be seen in Figure 10. Those with the lowest value correspond to the extracts obtained using the maceration method, and those with the highest value correspond to the extraction process carried out with Soxhlet and the ultrasonic bath. There is statistical homogeneity between the values obtained for both processes, with a value of 17.795 ± 0.0644 and 17.665 ± 0.0042a mg QE/g of dry sample of the extract obtained from the corresponding material dried at 40°C. In their study, Cortes-Rojas *et al*. ^34^ determined the flavonoid content of *Bidens pilosa* L. extracts by employing dynamic maceration, Soxhlet, ultrasound and microwave, obtaining corresponding values of 21.988 ± 0.127; 19.623 ± 0.100; 16.482 ± 0.180 and 11.590 ± 0.042 mg QE/g dry sample. These values coincide with those obtained in the present study, with the exception of those for dynamic maceration and microwave.

The equivalent mg of quercetin corresponding to each gram of dry *Croton floccosus* sample were obtained in the same way, as shown in Table 8. Tukey’s HSD test for the flavonoid content of the different samples determined that there is a significant difference among them (p <0.05). Samples that shared the same letter are homogeneous.

**Table 8.** Total flavonoid content corresponding to quercetin equivalents.

Sample	mg quercetin equivalent per g of dry sample			Average ± D. standard
	1	2	3	
Soxhlet at 40°C	34.117	34.209	34.091	34.139 ± 0.06224 ^a^
Soxhlet at 50°C	13.079	13.103	13.183	13.122 ± 0.05441 ^b^
Ultrasonic bath at 40°C	7.458	7.193	7.201	7.284 ± 0.15096 ^c^
Ultrasonic bath at 50°C	12.494	12.520	12.515	12.509 ± 0.01360 ^d^
Maceration at 40°C	22.201	22.281	22.329	22.270 ± 0.06461 ^e^
Maceration at 50°C	27.315	27.025	26.952	27.097 ± 0.1984 ^f^

Values are the mean of triplicate determinations. Different lowercase letters indicate statistical difference p<0.05.

Based on data from Ruiz Reyes *et al*. ^30^

**Figure 11.**
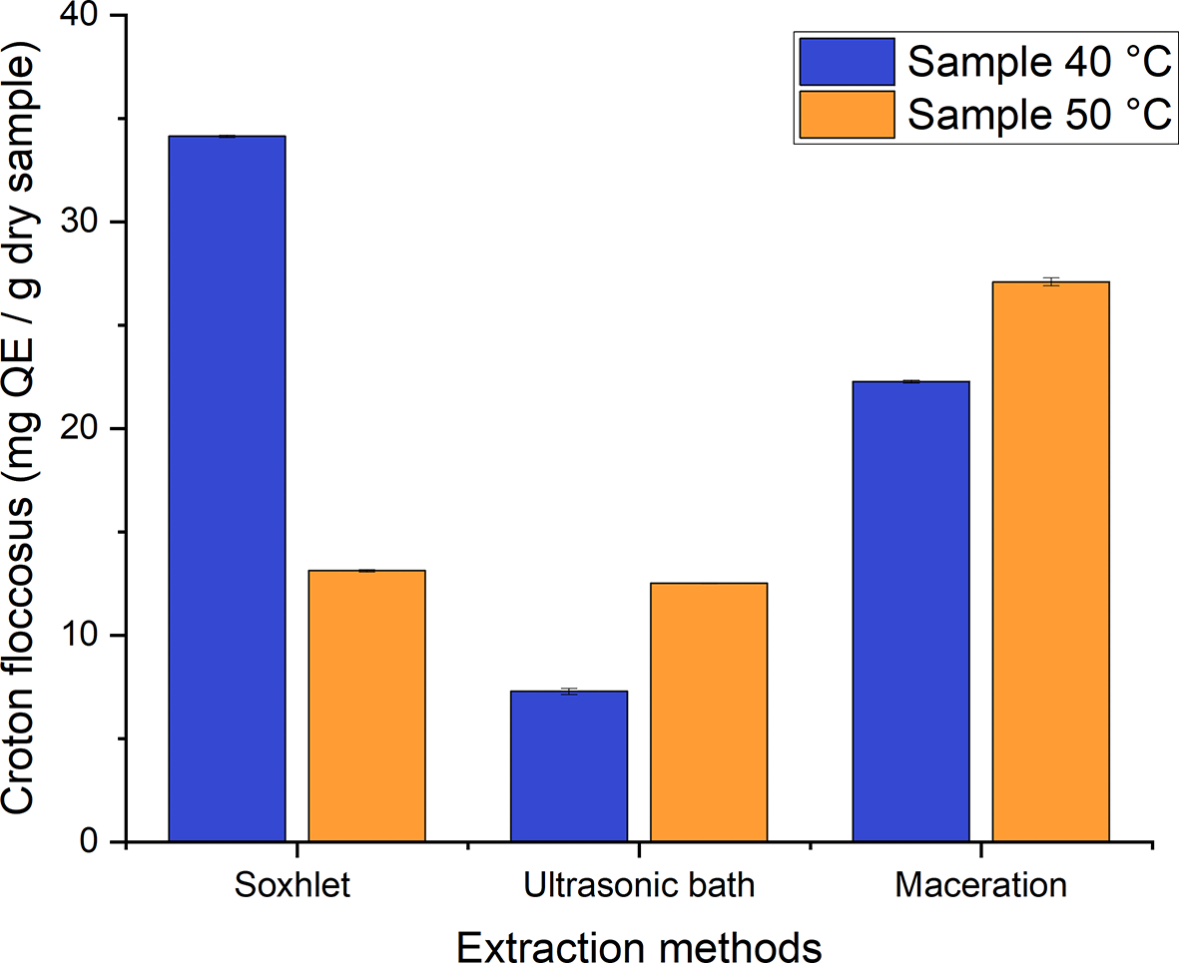
Total flavonoid content of the different ethanolic extracts of *Croton floccosus.*

The established range of total flavonoids comprises values between 7–35 mg QE/g of dry samples for the different processes used to obtain ethanolic extracts of *Croton floccosus* ( Figure 11). As can be seen, there is a great difference between the different treatments. That with the highest flavonoid content corresponds to the Soxhlet extraction process of the material dried at 40°C, with a value of 34.139 ± 0.06224 mg QE/g, while that with the lowest value corresponds to the extracts obtained by employing the ultrasonic bath method at the same temperature, with a value of 7.284 ± 0.15096 mg QE/g of dry sample.

### Antioxidant capacity (DPPH)

**Figure 12.**
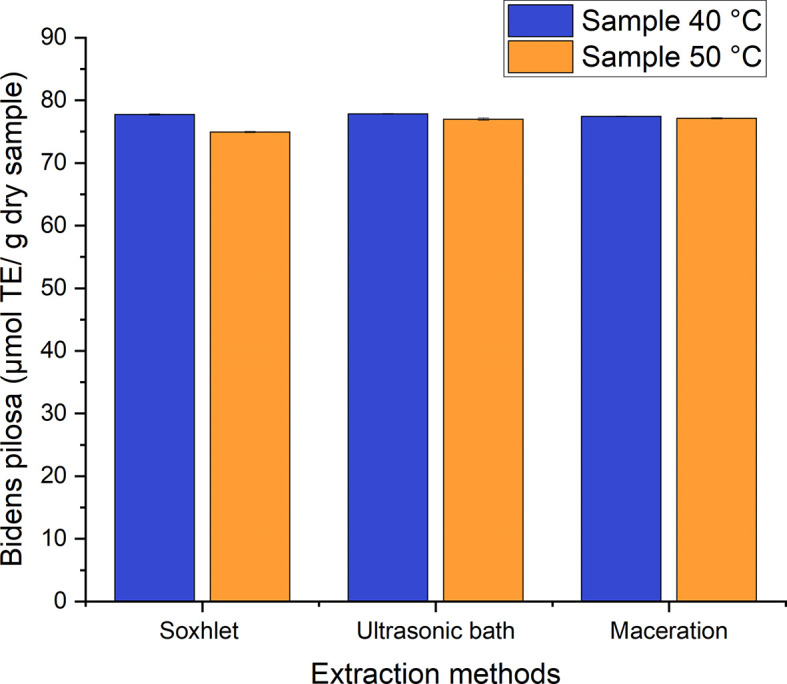
Antioxidant activity equivalent to Trolox of the different extracts of *Bidens pilosa* L.

The *in vitro* antioxidant activity was performed by employing the 2.2-diphenyl-1-picryl-hydracil radical scavenging method (DPPH assay). All the antioxidant activity values for the ethanolic extracts of both species were obtained from the Trolox equivalent calibration curve (TEAC). The established range for the Trolox equivalent antioxidant activity (TEAC) for *Bidens pilosa* L. is 74–78 μmol Trolox equivalent (TE)/g of dry samples for the different processes used to obtain ethanolic extracts with leaves dried at 40°C and 50°C ( Figure 12). The means of the values were compared using the Tukey test, which showed that there is no marked difference among the processes studied with regard to the TEAC. It was, therefore, established that the drying temperatures do not directly influence the TEAC values for the maceration, ultrasonic bath and Soxhlet extraction processes.

Considering that there was no marked difference among the processes, we then proceeded to establish the mean radical inhibition coefficient (IC50) of the process that attained the highest antioxidant activity value equivalent to Trolox, Soxhlet extraction of the material dried at 50°C. Table 9 shows the inhibition coefficient (IC50) determined by the DPPH method performed on the extract of *Bidens pilosa* L. and Trolox (standard). The difference between the extract and the standard used in the previous determinations is compared.

**Table 9.** Comparison of inhibition coefficients for *Bidens pilosa* L. and Trolox.

Sample	IC _50_ (μg/mL)
*Bidens pilosa* L. ethanolic extract	239.333
Trolox	2.599

The values reported in the present study are lower than those reported by Cortés-Rojas *et al*., ^34^ who obtained an IC50 for the DPPH radical of 35.35 μg/mL for *Bidens pilosa* L. extracts from flowers and leaves collected in Brazil using dynamic maceration at 45°C and with 200 rpm of agitation. Furthermore, Singh *et al*. ^31^ obtained an IC50 value of 80.45 μg/mL for extracts from the leaves of the same species collected in India using maceration with methanol for 48 h. We are, therefore, of the opinion that the differences between our IC50 values and those reported are related to the time of year and the country in which the plant was collected, along with the conditions under which the plant material was prepared and the method used to obtain the extract of secondary metabolites.

**Figure 13.**
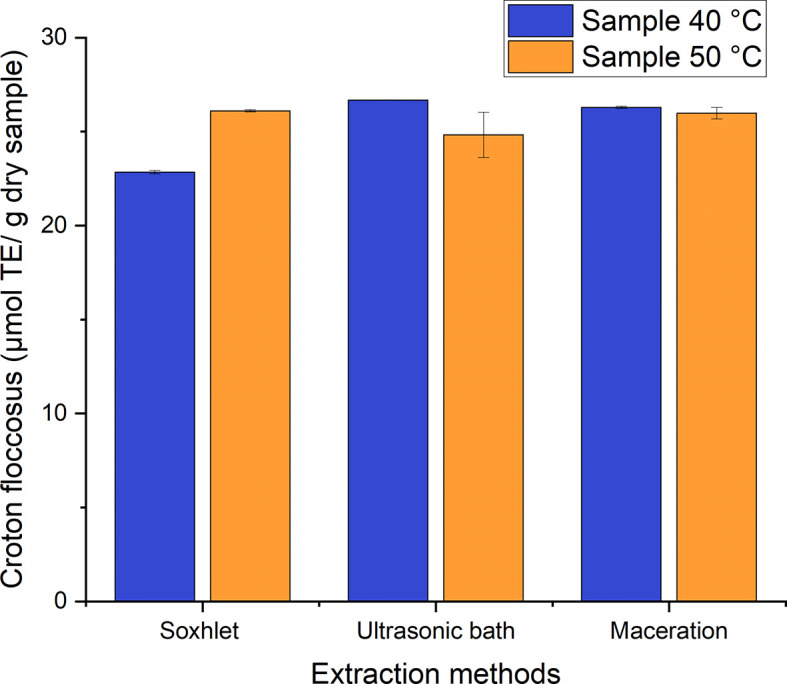
Antioxidant activity equivalent to Trolox of *Croton floccosus.*

The established range for the Trolox equivalent antioxidant activity (TEAC) for *Croton floccosus* is 22–27 μmol TE/g of dry samples for the different processes used to obtain ethanolic extracts with leaves dried at 40°C and 50°C ( Figure 13). The means of the values were compared using the Tukey test. No marked difference among the processes studied was found for the TEAC, and there was statistical homogeneity among them. It was, therefore, established that, as with *Bidens pilosa* L., there is no marked difference in the antioxidant activity values obtained using the different extraction processes carried out in the present research.

As with the previous process, there is no marked difference among the methods, and we, therefore, proceeded to establish the mean radical inhibition coefficient (IC50) of the process that obtained the highest value of antioxidant activity equivalent to Trolox, which was the ultrasound of the material dried at 40°C.

Table 10 shows the inhibition coefficient (IC50) determined by the DPPH method performed on the extract of *Croton floccosus* and Trolox (standard). The difference between the extract and the standard used in the previous determinations is compared.

**Table 10.** Comparison of inhibition coefficients for *Croton floccosus* and Trolox.

Sample	IC _50_ (μg/mL)
*Croton floccosus* ethanolic extract	644.125
Trolox	2.599

It is necessary to mention that, since *Croton floccosus* is a species that is endemic to Ecuador, there are only a few studies referring to this species. In his study, M.E. Flores ^35^ obtained IC50 values of 91.16 μg/mL for extracts obtained from the leaves of *Croton floccosus* collected during the flowering period of this species using a maceration process in which ethanol was employed as a solvent. This, therefore, made it possible to establish what is described by Altamirano ^36^ in his research work on Croton species, which states that the collection carried out to obtain extracts must take place at the beginning of flowering because it is the moment at which these plants contain the greatest amount of active substances. This may, therefore, have influenced the properties of the extract, as may the conditions under which the material was prepared, the extraction and the storage of the extracts.

## Conclusions

The use of phytochemical tests to identify metabolites, along with their qualitative characterization show that the plants studied have a great variety of chemical compounds, which contain flavonoids, phenols, reducing sugars, saponins and tannins in the case of *Bidens pilosa* L., and alkaloids, phenols, flavonoids, tannins and saponins in that of *Croton floccosus.* It has been shown that the content of total phenols for *Bidens pilosa* L. had the highest yield of phenolic compounds when the Soxhlet extraction method was used with the plant material dried at 50°C, with a value corresponding to 48.609 ± 0.370 mg GAE/g of dry sample. With regard to the content of flavonoids, a maximum value of 17.795 ± 0.0644 mg QE/g of dry sample was obtained, corresponding to the Soxhlet extraction process method at 40°C. In the case of *Croton floccosus*, the maximum reported value of total phenols corresponds to the maceration extraction process when the material had been dried at 50°C, with a value of 128.212 ± 0.601 mg GAE/g of dry sample. With regard to the content of total flavonoids, the best results corresponded to the extraction process carried out using Soxhlet extraction with the plant material dried at 40°C, with a value of 34.139 ± 0.06224 mg QE/g dry sample. Finally, both plant species showed antioxidant potential, although it is necessary to establish that the *Bidens pilosa* L. species responded better to the test established, with an IC50 of 239.33 μg/mL when compared to *Croton floccosus*, which attained an IC50 of 644.125 μg/mL.

## Data availability

### Underlying data

Open Science Framework: Underlying data for ‘Phytochemical study of the plant species *Bidens pilosa* L. (Asteraceae) and *Croton floccosus* (Euphorbiaceae)’, https://doi.org/10.17605/OSF.IO/VNPMJ. ^30^

This project contains the following underlying data:

• Data file: Antioxidant activity DPPH.xlsx
• Data file: Flavonoids.xlsx
• Data file: Phenols.xlsx

Data are available under the terms of the Creative Commons Zero “No rights reserved” data waiver (CC0 1.0 Public domain dedication).
