# Supplementary material for: Phytochemical study of the plant species Bidens pilosa L. (Asteraceae) and Croton floccosus (Euphorbiaceae)
Source: F1000Res. 2022 Oct 17;11:702. Originally published 2022 Jun 27. [Version 2] doi: 10.12688/f1000research.112653.2 (PMC9623199; doi:10.12688/f1000research.112653.2)
Supplement: Peer review history

# Peer review: Reviewer response for version 2

**DOI:** 10.5256/f1000research.139483.r153490

**Published:** 2022-10-31

**Authors:** Pawel Konieczynski

**Affiliations:** 1 Department of Analytical Chemistry, Medical University of Gdansk, Gdansk, Poland

Dear Authors,

 I appreciate the changes you have made to the manuscript. I am glad that my comments were taken into consideration and now the improved article is better. I have no other questions or comments.

Is the work clearly and accurately presented and does it cite the current literature?

Yes

If applicable, is the statistical analysis and its interpretation appropriate?

Yes

Are all the source data underlying the results available to ensure full reproducibility?

Partly

Is the study design appropriate and is the work technically sound?

Yes

Are the conclusions drawn adequately supported by the results?

Yes

Are sufficient details of methods and analysis provided to allow replication by others?

Partly

Reviewer Expertise:

Analytical chemistry

I confirm that I have read this submission and believe that I have an appropriate level of expertise to confirm that it is of an acceptable scientific standard.

# Peer review: Reviewer response for version 2

**DOI:** 10.5256/f1000research.139483.r153489

**Published:** 2022-10-24

**Authors:** Yailet Albernas Carvajal

**Affiliations:** 1 Chemical Engineering Department, Chemistry and Pharmacy Faculty, Central University “Marta Abreu” of Las Villas, Santa Clara, Villa Clara, Cuba

Paper is correct.

Is the work clearly and accurately presented and does it cite the current literature?

Partly

If applicable, is the statistical analysis and its interpretation appropriate?

I cannot comment. A qualified statistician is required.

Are all the source data underlying the results available to ensure full reproducibility?

Yes

Is the study design appropriate and is the work technically sound?

Yes

Are the conclusions drawn adequately supported by the results?

Yes

Are sufficient details of methods and analysis provided to allow replication by others?

Yes

Reviewer Expertise:

Chemical Engineering

I confirm that I have read this submission and believe that I have an appropriate level of expertise to confirm that it is of an acceptable scientific standard.

# Response

**Published:** 2022-10-24

**Authors:** Enrique Ruiz Reyes

Dear PhD. Yailet Albernas Carvajal, I am grateful for the analysis of version 2 of the article that you have carried out.

# Peer review: Reviewer response for version 1

**DOI:** 10.5256/f1000research.124381.r151932

**Published:** 2022-10-04

**Authors:** Yailet Albernas Carvajal

**Affiliations:** 1 Chemical Engineering Department, Chemistry and Pharmacy Faculty, Central University “Marta Abreu” of Las Villas, Santa Clara, Villa Clara, Cuba

The article entitled "Phytochemical study of the plant species *Bidens pilosa* L. (Asteraceae) and *Croton floccosus* (Euphorbiaceae)" is very interesting because it performs a preliminary phytochemical study of the plants *Bidens pilosa* L. and *Croton floccosus*. Important information was determined related to the content of secondary metabolites and the response to the DPPH radical reported for the first time in Ecuador, a relevant aspect, which may be of future use for its application in the field of medicine. They show that both plants studied have antioxidant potential, although they state that it is necessary to establish that the species *Bidens pilosa* L. responded better to the established test, with an IC50 of 239.33 μg/mL compared to *Croton floccosus*, which reached an IC50 of 644.125 μg/mL.

 The methods employed are very well supported with adequate techniques, using modern analytical equipment and techniques. It presents a correct description as to be replicable should any reader need it with any other species.

**Minor point:**

- Incorporate other references from the last three years.

Is the work clearly and accurately presented and does it cite the current literature?

Partly

If applicable, is the statistical analysis and its interpretation appropriate?

I cannot comment. A qualified statistician is required.

Are all the source data underlying the results available to ensure full reproducibility?

Yes

Is the study design appropriate and is the work technically sound?

Yes

Are the conclusions drawn adequately supported by the results?

Yes

Are sufficient details of methods and analysis provided to allow replication by others?

Yes

Reviewer Expertise:

Chemical Engineering

I confirm that I have read this submission and believe that I have an appropriate level of expertise to confirm that it is of an acceptable scientific standard.

# Response

**Published:** 2022-10-05

**Authors:** Enrique Ruiz Reyes

I agree with the evaluation issued by the reviewer and, following their suggestion, references 24, 25 and 26 were replaced by others that correspond to the last 3 years.

# Peer review: Reviewer response for version 1

**DOI:** 10.5256/f1000research.124381.r142422

**Published:** 2022-07-19

**Authors:** Pawel Konieczynski

**Affiliations:** 1 Department of Analytical Chemistry, Medical University of Gdansk, Gdansk, Poland

I appreciate the work entitled: Phytochemical study of the plant species *Bidens pilosa* L. (Asteraceae) and *Croton floccosus* (Euphorbiaceae). The strengths of the manuscript can be listed below:

1. The Authors have thoroughly studied two important folk medicine plants originating from two botanical species growing in Ecuador.
2. They investigated total content of phenols and flavonoids as well as the antioxidant activity using DPPH method for comparison of two plants.
3. The choice of materials and methods were appropriate, not counting my minor comments given below.
4. The statistics applied for interpretation of the experimental data is correct.
5. The conclusions drawn by the Authors are justified by their results.

**Minor comments**

1. Page 3: in Methods (Vegetal material) - please write whether the fresh leaves of the studied plant species were collected only from one plant or from several plants, and then mixed to create the sample for analysis?
2. Page 3: in Extraction - please add more details on how the sample was pulverized (for example what type of sample mill was used, how long it lasted)?
3. I also think that it would be helpful to add a map showing the collection places in Portoviejo Botanical Garden.

Is the work clearly and accurately presented and does it cite the current literature?

Yes

If applicable, is the statistical analysis and its interpretation appropriate?

Yes

Are all the source data underlying the results available to ensure full reproducibility?

Partly

Is the study design appropriate and is the work technically sound?

Yes

Are the conclusions drawn adequately supported by the results?

Yes

Are sufficient details of methods and analysis provided to allow replication by others?

Partly

Reviewer Expertise:

Analytical chemistry

I confirm that I have read this submission and believe that I have an appropriate level of expertise to confirm that it is of an acceptable scientific standard, however I have significant reservations, as outlined above.

# Response

**Published:** 2022-07-21

**Authors:** Enrique Ruiz Reyes

1 - Referee's first comment:

 The leaves that present better conservation in their structure are chosen to clean them of dust and branches, then the petiole of the leaf blade is extracted. The studied leaves were collected from various plants and then mixed to create the sample for analysis.

 2 - Referee's second comment:

 The sample was pulverized using a blade mill (grinder) with a sieve so that a more uniform size of the material can be obtained, in order to fractionate the plant tissue and produce a better extraction. The time used for grinding was 5 minutes.

 3 - Referee's third comment:

 It is shown in the following map of the botanical garden. The collection points of the studied species. For *croton floccosus* it was two points while for *bidens pilosa* it was three points. The coordinates of the studied species are placed on the map, which is linked here.
